# “Flexible nature of fixation” in syndesmotic stabilization of the inferior tibiofibular joint affects the radiological reduction outcome

**DOI:** 10.1007/s00264-022-05550-7

**Published:** 2022-08-19

**Authors:** Robert Hennings, Carolin Fuchs, Ulrich J. Spiegl, Jan Theopold, Firas Souleiman, Christian Kleber, Annette B. Ahrberg

**Affiliations:** Department of Orthopaedics, Traumatology and Plastic Surgery, University of Leipzig, Liebigstr. 20, 04103 Leipzig, Saxony Germany

**Keywords:** Inferior tibiofibular joint, Syndesmosis, Suture button, Flexible fixation, Ankle

## Abstract

**Purpose:**

Fibular mobility after suture button stabilization (SBS) of inferior tibiofibular joint **(**syndesmosis) injuries has been described. This effect is called the “flexible nature of fixation (FNF).” In this study, we aimed to quantify FNF in syndesmotic stabilization.

**Methods:**

Postoperative bilateral computed tomography (CT) of ankle fractures with syndesmosis stabilization by SBS or syndesmotic screw (SYS) was retrospectively analyzed. The transverse offset (TO) and vertical offset (VO) were defined by evaluating the drill channels. The reduction outcome was evaluated by the side-to-side difference between the clear space and the anterior tibiofibular distance (antTFD). The calculated anterior tibiofibular distance (cal-antTFD) was calculated by subtracting the TO from the validated antTFD. Subsequently, a reevaluation of the reduction outcomes after SYS or SBS stabilization was performed using cal-antTFD.

**Results:**

Sixty patients (44 with SBS and 16 with SYS stabilization) were analyzed. The intra-rater and inter-rater reliabilities for TO and VO were excellent (*α* > 0.92). SYS stabilization showed lower mean TO (− 0.02 mm; SD 0.14) and VO (0.11 mm; SD, 0.29 mm) than SBS stabilization (TO 1.16 mm, SD 1.4 mm; VO 0.2 mm, SD 0.8 mm; *p* = 0.001). The rate of malreduction according to cal-antTFD was higher than that of antFTD (*p* = 0.033).

**Conclusion:**

The presented method, which evaluates the position of the tibial to the fibular drill channel, allowed the quantification of the “FNF.” The often described difference in the dynamic stabilization of SBS compared to the rigid stabilization by SYS could be objectified. Considering cal-antTFD illustrates that FNF potentially reduces the rate of malreduction in SBS stabilization.

## Introduction

Injury of the inferior tibiofibular joint (syndesmosis) can occur isolated or in ankle fractures [1–6]. There is a broad consensus that anatomic reduction and stabilization should be performed due to better clinical outcomes and prevention of secondary degenerative changes [7–10]. In addition to stabilization using a static syndesmotic screw (SYS), which has been performed for decades, stabilization with a suture button system (SBS) was introduced in the 2000s [11, 12]. This has proven to be an effective method for stabilizing isolated as well as fracture associated syndesmosis injuries and has become more established in recent years [4, 5, 13–16]. SBS tends to lower the rates of malreduction and results in better early postoperative functional outcomes; however, which method should be preferred has not yet been conclusively clarified [5, 10, 15, 17–23].

Studies have shown that the fibula moves three-dimensionally in response to foot rotation [14, 24–26]. In the case of deliberate syndesmotic malreduction in a cadaver model, suture button fixation resulted in less post-fixation displacement than SYS fixation [27]. Furthermore, case reports showed different positions of the fibula in the tibial incisura when comparing intra-operative with post-operative computed tomography (CT) or divergent tibial and fibular drill channels within suture button stabilization [16, 28]. Improved syndesmotic congruency has been described one year after stabilization with SBS, especially in posterior malreduction [29]. According to the authors, these differences are signs of existing fibular mobility after stabilization using the SBS. This effect is referred to as the “flexible nature of fixation” (FNF) or dynamic stabilization [16, 27, 28]. Several authors have demonstrated the advantage of suture button stabilization compared to SYS [16, 27–29].

Nevertheless, there are only a few in vivo studies that attempt to objectify the flexible, dynamic nature of fixation [26, 27]. Therefore, in this study, we aimed to quantify “FNF” in patients with stabilized syndesmosis based on the null hypothesis of no effect of FNF on post-operative reduction of the distal tibiofibular joint.

## Materials and methods

Approval of the local institutional review board was given beforehand (AZ 488/19-ek), and the study was conducted in accordance with the Declaration of Helsinki and the guidelines for Good Clinical Practice.

Consecutive adult patients who underwent surgical stabilization of the syndesmosis with SYS or SBS following an AO (Arbeitsgemeinschaft für Osteosynthesefragen) 44 B or 44 C fracture between 01/2010 and 12/2019 were included. The inclusion and exclusion criteria are summarized in Table 1 [30, 31]. Patient data were stored in an electronic database using SPSS (IBM, Version 24, Chicago, IL, USA).

**Table 1:** Inclusion and exclusion criteria. *CT*, computed tomography

Inclusion criteria	Exclusion criteria
• AO 44 B or 44 C fracture • Unstable syndesmosis • Unilateral stabilization of syndesmosis with SBS • Uninjured ankle without pathology • Postoperative bilateral CT control • CT slice thickness ≤ 1.0 mm • Anatomic reduction of all fracture components	• Age < 18 years • Bilateral ankle and/or syndesmosis lesions • Pathology of the uninjured ankle • Non-anatomic reduction of fractures with bone steps > 2 mm

### Operative management

Informed consent was obtained from all patients before surgery; patients were treated with a similar operative procedure according to the recommendations of the AO [30, 32]. All operations were performed by experienced specialists in trauma surgery of a trauma level I centre. If not evident on pre-operative imaging, syndesmotic instability was evaluated by standard fluoroscopy (lateral and mortise views) following fracture stabilization using the hook test with the ankle placed in a neutral dorsiflexed position [30, 32, 33]. When instability was detected, syndesmosis reduction was performed under direct visualization and fluoroscopy with reduction forceps placed in line with the transmalleolar axis of the neutral dorsiflexed positioned foot. Next, with the foot in neutral position, preliminary fixation is performed with a K-wire placed at an angle of approximately 30° from posterior to anterior parallel to the tibial plafond [32]. If the congruency on fluoroscopy in two planes was evaluated as anatomical, stabilization followed with a suture button device (TightRope®, Arthrex, Naples, FL, USA) or an SYS (3.5 mm, DePuy-Synthes, USA) as preferred by the surgeon [11, 32]. Reduction and fixation were controlled intra-operatively by fluoroscopy. Studies have shown that advanced age and osteoporosis are risk factors for adverse events after syndesmotic fixation [17]. Therefore, if osteoporosis was known or suspected based on the surgeon’s assessment of bone quality or trauma mechanism, SYS were used according our hospital’s standard [17, 32].

### Imaging

All CT scans were obtained during the inpatient period without intravenous contrast medium administration as part of standard care to assess syndesmotic reduction. The patients were positioned in a supine position and feet first, with the ankle in a neutral position. Images were acquired using a multidetector CT scanner (iCT 256; Philips, Netherlands). Routine scan parameters included a tube current of 150 mA, tube voltage of 100 kV, and collimation of 64 × 0.625 mm. The pitch was 0.329 with a rotation time of 0.5 s. Multiplanar reformations were reconstructed in slice thicknesses of 0.67 − 1.0 mm in axial, sagittal, and coronal orientation.

### Assessment and measurement of syndesmotic parameters

**Fig. 1:**
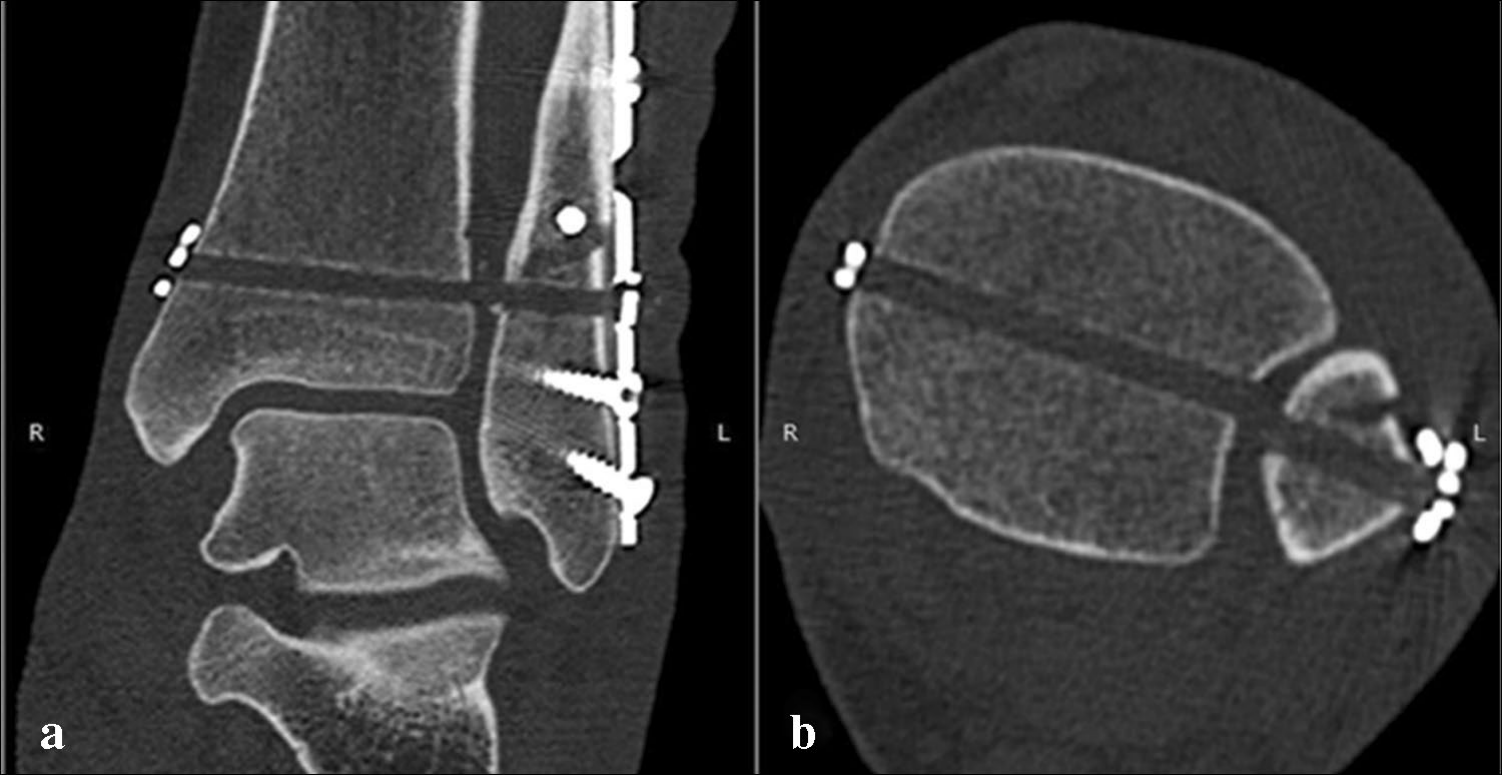
Computed tomography in axial (**a**) and coronal (**b**) reconstruction of a left ankle fracture classified as AO 44B3.1 after open reduction and internal fixation of the lateral malleolus (lag screw and neutralization plate) and dynamic stabilization of the distal tibiofibular joint

Standardized measurements were independently performed by one subspecialist foot and ankle surgeon (RH) and one resident (CF) using the RadiAnt DICOM Viewer 2020.2.3 (Medixant, Poznań Poland). The observers were trained in the measurement methods and performed twice, four weeks apart. First, all CT scans were aligned in the sagittal plane according to the longitudinal axis of the tibia (Fig. 1a–c). For complete visualization of the device, both the transverse and coronal planes were adjusted along the longitudinal axis of the stabilization device (Fig. 1).

### Measurement of transverse offset (TO) of the device (Fig. 2a)

**Fig. 2:**
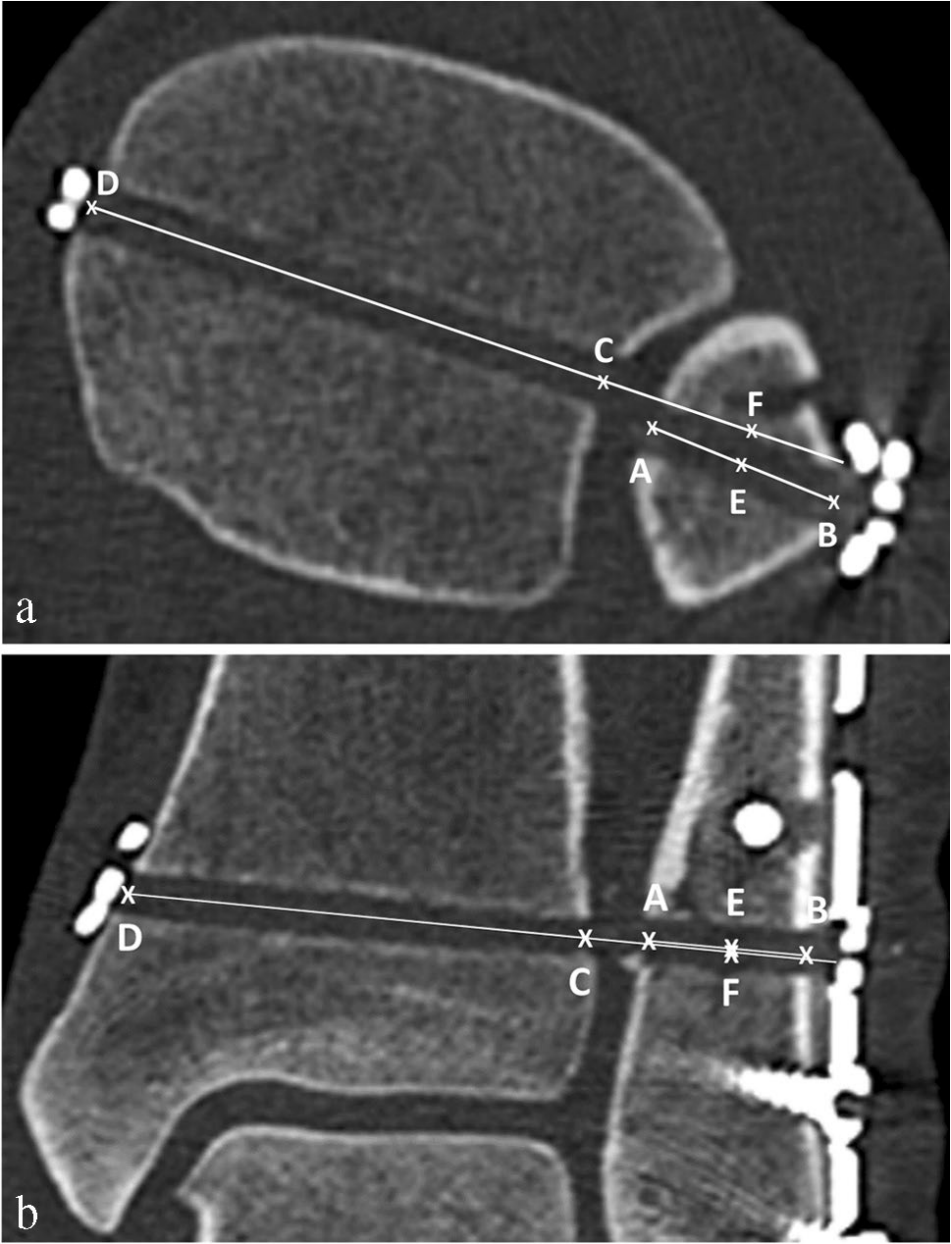
Axial (**a**) and coronal (**b**) computed tomography reconstruction of a left ankle after open reduction and internal fixation of the lateral malleolus (plate) with anatomical dynamic stabilization of the distal tibiofibular joint, lateral endobutton, and medial flip anchor. a Assessment of the axial plane: A–B fibular line, C–D tibial line, E–F transversal offset (TO). b Assessment of the coronal plane: A–B fibular line, C–D tibial line, distance E–F = vertical offset (VO)

First, the centres of the drill channel of the fibula (A and B) and tibia (C and D) were marked in line with the cortical bone. The fibula line was then drawn from points A to B, and the tibia line was drawn from point D to point C. The tibial line was extended to point B on the fibular side. Thereafter, the midpoint of the fibular line E was marked, and the measured distance from C to E was marked on the fibular side of point C on the tibial line as point F.

The distance measured between E and F represents the TO. A positive TO represents an assumed posterior displacement, and a negative TO represents the assumed anterior displacement of the fibular drill channel to the tibia.

### Measurement of vertical offset (VO) of the device (Fig. 2b)

Similarly, the centers of the drill channel of the fibula (A and B) and tibia (C and D) were marked in line with the cortical bone. The fibula line (A to B) and the tibia line (D to C), which was extended to the level of point B on the fibular side, were drawn. Following distances along the perpendicular line of the midpoint of lines A–B and the intersection of the extended tibial line describe the VO. A positive VO describes an assumed cranialization of the fibular drill channel, or a negative VO describes a distalization of the fibula to the tibia.

### Analysis of the reduction results

First, the CT planes were aligned with the longitudinal and sagittal anatomic axes of the tibia and the transmalleolar axis. To evaluate syndesmotic reduction, Leporjärvi Clear Space (LCS) was used to analyze the mediolateral translation and the anterior tibiofibular distance according to Ahrberg for sagittal alignment (antTFD) 10 mm proximal to the plafond [34–36]. These parameters were selected due to their high intra-observer and inter-observer reliabilities [34, 35]. The side-to-side difference between the injured and uninjured sides was calculated and defined as ΔLCS and ΔantTFD. A positive ΔLCS represents a widening of the syndesmosis, while positive ΔantTFD was defined as a posterior translation of the fibula in relation to the tibia on the injured side. An asymmetrical congruity |ΔLCS|> 2 mm and |ΔantTFD| of > 2 mm were evaluated as malreduction following the literature [7, 37, 38].

### Theoretical consideration of the reduction results

It was assumed that the reduction was temporarily fixed before stabilization and that the tibial and fibular lines did not deviate during the drilling and insertion of the SBS.

**Fig. 3:**
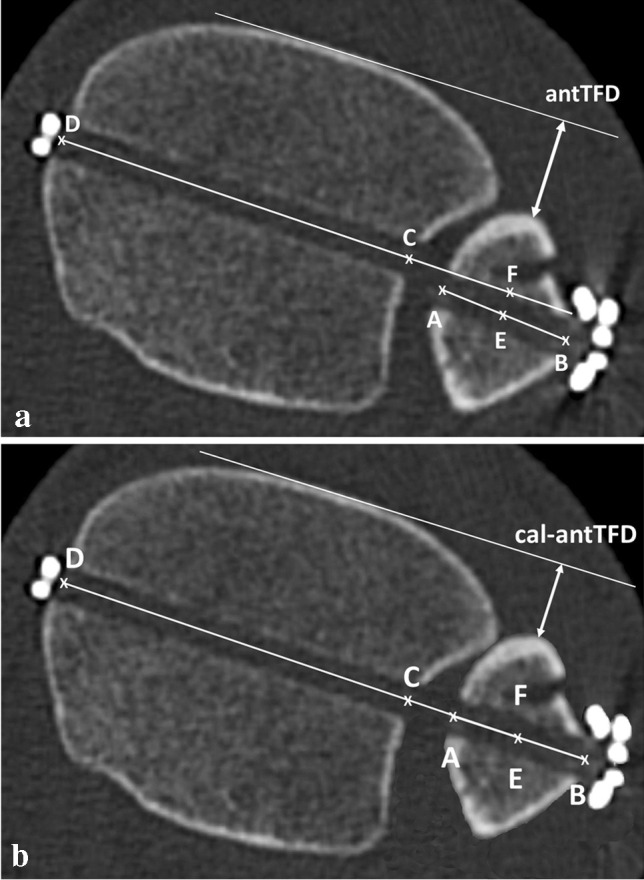
**a** Schematic representation of the measurement of the anterior tibiofibular distance (antTFD). **b** Illustration of the calculated tibiofibular distance (cal-antTFD) and the assumed tibiofibular relations after computational neutralization of TO (distance E–F) by subtracting TO from antTFD with the image processing program Gimp (GNU Image Manipulation Program, V 2.10.20). The distance C–E was rotated on C until the points E and F overlapped. The distance C–E is the same as that of C–F

The calculated anterior tibiofibular distance (cal-antTFD) was obtained by subtracting the TO from the validated antTFD. Thus, the deviation of the tibia and fibula lines was mathematically eliminated (Fig. 3a, b). Subsequently, the side-to-side difference of cal-antTFD and uninjured sides was calculated (Δcal-antTFD), and the reduction results were reevaluated as described above. An asymmetrical congruity |Δcal-antTFD| of > 2 mm was evaluated as malreduction.

### Statistics

Statistical analysis was performed using SPSS software (Version 25, IBM, Chicago, IL, USA). Student’s *t* test or Mann − Whitney *U* test was used to compare continuous variables between the two study groups depending on normal distribution and study size (Shapiro–Wilk test). Categorical variables were compared using Pearson’s chi-square test or Fisher’s exact test. Statistical significance was set at *p* ≤ 0.05. Cronbach’s alpha (*α*) was used to analyze the inter-rater and intra-rater reliabilities and was interpreted as acceptable (*α* < 0.70), good (*α* < 0.80), or excellent (*α* > 0.90) [39].

## Results

Sixty patients (mean age 44 years, range 18 to 84 years, SD 17 years) of the 184 patients who underwent stabilization of syndesmosis during the study period met the inclusion criteria. Of these, 44 patients with a mean age of 39 years (SD 14 years) were stabilized with SBS (SBS group). Sixteen patients with a mean age of 55 years (SD 18 years) were stabilized using SYS (SYS group). Patients stabilized with SBS were younger than those with SYS (*p* = 0.001), while the sex distribution of the two groups did not differ (*p* = 0,785; Table 2).

**Table 2:** Cronbach’s alpha of inter-rater and intra-rater reliability analyses

	*Cronbach’s alpha*	
	Transversal offset	Coronal offset
Intra-rater RH	0.99	0.94
Intra-rater CF	0.99	0.93
Inter-rater	0.98	0.92

### Parameters describing “FNF”

The mean TO of the SBS group was 1.2 mm (SD, 1.4 mm). The mean AD was 7.8° (range, − 13° − 45°). The mean of vertical offset (VO) was − 0.2 mm (SD, 0.8 mm; Table 1). No differences were seen between sexes for all three parameters (*p* > 0.005 each). Thereby, the SYS group showed significant lower mean of TO (− 0.26 mm; SD, 0.18 mm) and VO (0.11 mm; SD, 0.29 mm; *p* = 0.001; Table 1) than the SBS group. A deviation of the fibular to the tibial drill channel in the axial plane was observed in 95% of the cases with SBS stabilization. In contrast, this was only observed in three cases after SYS (18%).

Both the intra-rater and inter-rater reliabilities for parameters describing the “FNF” were consistently excellent for each measurement (*α* > 0.90, Table 2).

### Relationship between the parameters describing FNF and reduction outcome

According to the ΔantTFD, eight out of 44 patients (18%) in the SBS group and six out of 16 patients (37.5%) in the SYS group were evaluated as malreduced, with no difference between the two groups (*p* = 0.186). In the SBS group, patients with posterior malreduction tended to have greater TO than those with anterior malreduction or anatomic reduction (*p* = 0.06, Table 3).

**Table 3:** Characteristics of the patients, fracture pattern, and comparison of both stabilization procedures. All data are presented as mean (SD; range). *Δ*, side-to-side difference; *antTFD*, anterior tibiofibular distance; *cal-ΔantTFD*, theoretical anterior tibiofibular distance. ^a^posterior malleolus without fixation, ^b^posterior malleolus fixed

	All patients *N* = 60		*P*-value
	Females *N* = 28	Males *N* = 32	
Mean age (SD) in years	46.4 (18.3)	41.5 (15.3)	0.287
	Stabilization		
	SYS group *N* = 16	SBS group *N* = 44	
Mean age (SD, range) in years	55 (18; 23–83)	39 (14; 18–68)	**0.001**
Female: male	7: 9	21: 23	0.785
Right: left	5: 11	18: 26	0.496
Anatomy and osteosyntheses of fractures, *N*			
Isolated fibula	9	20	
Fibula and medial mall	2	6	
Fibula and posterior mall^a^		8	
Fibula, medial and posterior mall^a^	5	6	
Trimalleolar fracture^b^		4	
Transversal offset in mm Mean (SD; range)	− 0.02 (0.14; − 0.52–0.23)	1.16 (1.4; − 2.9–5.7)	0.001
Angulation of the device (°)			
Mean (SD; range)	0.2 (0.5; 0–2)	7.7 (10.8; − 12–45)	0.001
Coronal offset in mm			
Mean (SD, range)	0.08 (0.24; 0–0.93)	− 0.26 (0.76; − 4.0–0.95)	0.012
ΔantTFD in mm	0.16 (2.9; − 6.9–4.7)	0.42 (2.4; − 4.0–9.0)	0.728
cal-ΔantTFD in mm	− 0.21 (4.2; − 13–4.7)	− 0.74 (2.1; − 4.7–5.7)	0.514
ΔLCS	0.52 (1.2; − 1.9–4.20)	0.71 (0.9; − 0.8 to 2–19)	0.560

According to the ΔLCS, five out of 44 patients (11%) in the SBS group and one out of 16 patients (7%) in the SYS group were evaluated as diastasis, with no difference in means between the two groups (*p* = 0.560, Table 4). No overtightening of the syndesmosis was observed. TO of patients in the SBS group rated as anatomic or diastasis according ΔLCS did not differ (median 1.21 mm SD 1.48 vs median 0.61 mm SD 0.94; *p* = 0.260).

**Table 4:** Overview of the transversal offset (TO) of the different reduction result groups according to the real reduction (antTFD) and the calculated reduction (cal-antTFD)

Reduction outcome according to antTFD				*P*-value
	Anterior malreduced *N* = 4	Anatomical reduction *N* = 36	Posterior malreduced *N* = 4	
Transversal offset	0.6 (1.0)	1.0 (1.3)		0.499
(TO) in mm		1.0 (1.3)	3.0 (2.1)	0.060
	0.6 (1.0)		3.0 (2.1)	0.057
Calculated reduction outcome according to cal-antTFD				
	Spontaneous reduction of anterior malreduction *N* = 11	Persistent anterior malreduction *N* = 4	Persistent anatomic reduction *N* = 25	
TO in mm	1.9 (1.0) 1.9 (1.0)	0.6 (1.0)	0.6 (1.2)	0.259 0.006
cal-antTFD in mm	7.96 (2.7)	8.99 (2.0)		0.374
	7.96 (2.7)		11.52 (2.9)	0.001
ΔCS in mm	0.60 (1.05)		0.59 (1.26)	0.542

### Evaluation of the impact of FNF on the reduction

In the analysis according to the calculated cal-antTFD, 17 out of 44 patients (39%) stabilized with SBS were rated as malreduced. Of these, 15 patients were anterior, and two were posterior malreduced (Table 4). Analysis of cal-antTFD malreduction was found more often than in consideration of antTFD measurements in patients stabilized with SBS (*p* = 0.033).

In consideration of cal-ΔantTFD, 11 cases of assumed anterior malreduction could be shown to be evaluated as anatomically reduced due to a dorsal deviation of the fibula (Table 4). The largest cal-ΔantTFD in these patients was − 4.00 mm and was corrected to an anatomical position by FNF of 3.4 mm. In the comparison of the cal-ΔantTFD with ΔantTFD showed four patients had persistent anterior malreduction, two had persistent posterior malreduction, and two patients with dorsal malreduction on post-operative CT were considered anatomically reduced according to cal-antTFD. One patient of the computationally induced dorsal incongruity had an anatomically reduced Volkmann triangle and one had no fracture in this area.

The extent of TO and cal-antTFD in patients with spontaneous reduction of the calculated anterior malreduction (cal-ΔantTFD > 2 mm) by FNF and those in whom the anterior malreduction (cal-ΔantTFD > 2 mm) persisted did not differ (*p* = 0.163 and *p* = 0.336, respectively; Table 4). The cal-antTFD and TO of patients with spontaneous reduction in calculated anterior incongruity (cal-ΔantTFD > 2 mm) were greater than those with persistent anatomic reduction (*p* = 0.001 and *p* = 0.006, respectively). No differences in ΔCS of patients with spontaneous reduction in calculated anterior incongruity and persistent anatomic reduction was seen (Table 4, *p* = 0.896). In the context of stabilization with an SYS, the FNF did not show an effect on the rate of malreduction caused by the small amplitude of the FNF (*p* > 0.05; Table 2).

## Discussion

The objective of this study was to quantify the “FNF” in syndesmotic stabilization based on the null hypothesis of no impact on reduction results using an SYS or SBS. According to these data, there is a deviation of the fibula to the tibial drill channel in the axial plane in 95% of cases with SBS stabilization of the distal tibiofibular joint (syndesmosis). Malreduction was found more often according to the calculated cal-ΔantTFD than the real ΔantTFD measurements for SBS, but not for SYS. Therefore, the null hypothesis can be rejected for the SBS. Eleven assumed anterior malreductions were spontaneously corrected, but two posterior malformations were induced by FNF in this regard. In patients stabilized with static SYS, no effect on the rate of malreduction was observed. The results presented in this study objectified and quantified the presence of FNF in suture button stabilization of the syndesmosis described in cadaver studies and case reports [16, 26–28].

In evaluating the results and assessment of the tibial and fibula drill channels, we assume that they lie on the exact centre-line during drilling after reduction and temporary fixation, as described in the technical instructions [11]. The reduction of syndesmosis depends on the orientation of the reduction forceps, and the induced malreduction can be fixed subsequently by an SYS [27], whereby it has been shown that the orientation of syndesmosis fixative device does not affect immediate reduction [40]. The authors concluded that malreduction must be dedicated before the final fixation [40]. In this context, a modified glide path technique for syndesmotic reduction in ankle fracture fixation appears to reduce the rate of malreduction [41]. The static fixation of the syndesmosis by SYS was indicated by the lack of relevant divergence of the fibular drill channel to the tibial channel, as could have been assumed. The minimal deviation of the drill channels in the use of an SYS may be caused by minimal bending of the screw and by possible yielding of the cortical bone in the drill channel area. In cadaver studies, the extent of a defined malreduced syndesmosis fixed with reduction forceps during stabilization was lower in the sagittal direction after suture button fixation than after SYS [27]. Based on CT analyses, the congruency of the syndesmosis improved one year following SBS stabilization [29]. Our results support the explanation for these results due to the FNF of the SBS [27]. In agreement with Westermann et al., we attribute the presence of flexibility to the positioning variance of the suturing device within the drill channel due to the mismatch of the diameters of the drill channel and the suturing device [27]. However, the possibility of bending the suturing device within a clear space is a further explanation for the FNF. We assume that despite the presence of a suture device, the fibula can migrate in the direction of the natural concavity of the tibial incisura fibularis after removal of the reduction forceps, as also assumed by Westermann [27]. An effect of FNF on mediolateral congruity, especially on the increase of a diastasis, could not be detected in the results.

Our results also showed that the rate of malreduction in the sagittal plane was more frequent than in the coronary direction in the SYS and SBS [29, 38]. The rate of malreduction appears to be lower with the use of SBS than with SYS, which is considered a factor for tendentially better clinical outcomes after SBS [5, 15, 17, 27, 42–44]. Based on mathematical considerations, which are supported by the studies mentioned above, FNF might represent a possible approach to explain the trend towards better radiological and clinical outcomes after SBS [27, 29]. However, the results also indicated that not all cases of incongruence presented a sufficient reduction in the FNF. Remarkably, no differences in cal-antTFD were observed between patients with sufficient and insufficient calculated spontaneous reduction (Table 4). Also, in two patients with anatomic reduction according to cal-ΔantTFD, posterior malreduction was detected in the real assessment, although only anatomically reduced fractures were analyzed. The present study cannot provide an explanation for this. It would be possible that anatomical features promote malreduction, as has been shown for the syndesmosis screw [38]. Therefore, further studies are warranted to determine which additional factors contribute to FNF. Thus, an accurate reduction of syndesmosis is also essential when using an SBS to minimize the rate of malreduction. In addition, based on this study, visualization of syndesmosis is performed as far as possible through the chosen approach, and intra-operative control 3D CT is standard in our clinic.

In our opinion, the study could objectify and quantify the effect of FNF although there are some limitations that should be discussed. In addition to the retrospective design and the small sample size, the most important limitation is the assumption that the VO measured at the level of the suture button proportionally affects the parameters that describe the reduction, which are determined to be 10 mm above the tibial plafond. Therefore, the presentation of the effect of VO on reduction represents a calculated consideration. The surgeon’s selection of the stabilization technique is also a limitation, but in our opinion without an effect to the results. Furthermore, the results represent the “FNF” for using a single SBS to stabilize syndesmosis. Studies have shown that the use of a second SBS compared to one does not significantly influence the immediate reduction outcome [45, 46]. However, the extent to which the use of a second parallel or divergent SBS affects the FNF is still lacking. In view of this and the costs, which are lower in total than the use of a SYS and sequential material removal after 6 to 8 weeks, stabilization with one SBS is performed in our own center [22], whereby the routine removal of the SYS should be critically discussed [47]. Possible position-dependent measurement inaccuracies could be reduced by standardized positioning of the feet and exact adjustment of the CT planes but cannot be completely eliminated. FNF describes the dynamic aspects of SBS that are difficult to visualize using static imaging technology. An interesting question for further investigations is especially the influence of different rotations of the foot and load on the FNF analyzed with the presented method. There is also a controversy about the importance of immediate control by a weight-bearing CT [48, 49]. However, a follow-up one year after stabilization using a weight-bearing CT would be desirable.

Furthermore, the influence of fracture morphology on the parameters of interest was minimized by including only bony anatomically reduced fractures. Further results considering the influence of fracture morphology and interindividual anatomy are still pending in this regard. Furthermore, the analysis of the relationship between offset and clinical outcomes will also be a topic for further studies.

In conclusion, the method presented allowed the objectification and quantification the “FNF” by evaluating the position of the tibia in the fibular drill channel on CT. The often described difference in the dynamic stabilization of SBS compared to the rigid stabilization by SYS could be objectified. By considering the cal-ΔantTFD, it was shown that in suture button stabilization, the rate of malreduction may be reduced by the dynamic property. But exact adjustment of syndesmosis is also necessary for SBS stabilization, as not all anterior incongruities were adequately corrected by FNF, and in individual cases malreduction can be induced. The study raises further issues of interest, in particular which anatomical and fracture morphological factors influence FNF as well as to what extent it changes under loading and different foot positions.
